# The Vegetative State and Stem Cells: Therapeutic Considerations

**DOI:** 10.3389/fneur.2016.00118

**Published:** 2016-08-23

**Authors:** Alan S. Hazell

**Affiliations:** ^1^Department of Medicine, University of Montreal, Montreal, Quebec, Canada; ^2^Programa de Postgrado en Fisiopatología Médica, Universidade Estadual de Campinas (UNICAMP), Campinas, São Paulo, Brazil

**Keywords:** consciousness, neuroimaging, traumatic brain injury, hypoxia-ischemia, unresponsive wakefulness syndrome, stem cell, transplantation, induced pluripotent stem cell

## Abstract

The vegetative state (VS), also known as “unresponsive wakefulness syndrome,” is considered one of the most devastating outcomes of acquired brain injury. While diagnosis of this condition is generally well-defined clinically, patients often appear to be awake despite an absence of behavioral signs of awareness, which to the family can be confusing, leading them to believe the loved one is aware of their surroundings. This inequality of agreement can be very distressing. Currently, no cure for the VS is available; as a result, patients may remain in this condition for the rest of their lives, which in some cases amount to decades. Recent advances in stem cell approaches for the treatment of other neurological conditions may now provide an opportunity to intervene in this syndrome. This mini review will address the development of VS, its diagnosis, affected cerebral structures, and the underlying basis of how stem cells can offer therapeutic promise that would take advantage of the often long-term features associated with this maladie to effect a repair of the severely damaged circuitry. In addition, current limitations of this treatment strategy, including a lack of animal models, few long-term clinical studies that might identify benefits of stem cell treatment, and the potential for development of tumors are considered.

## Introduction

The diagnosis of “vegetative state” (VS) (1) assigned to patients following a severe injury to the brain is a devastating one. Originally, this condition was meant to indicate a complete lack of cortical functioning. However, a number of studies (2, 3) suggest that some VS patients possess at least elements of intact cognitive functions, e.g., pitch discrimination, or recognition of nonsensical sentence endings (3–6), and that following brain damage, some form of consciousness persists (7). Recently, VS has been termed an “unresponsive wakefulness syndrome” (8), in which patients may appear to be awake yet still not show behavioral signs of awareness. Indeed, some of these patients may start to show non-reflexive behavior, albeit inconsistently, e.g., gaze following, orienting responses, or command following. Such patients are then diagnosed as in a minimally conscious state (9). Correctly diagnosing these patients can be difficult, reflected in high rates of misdiagnoses (10).

In many countries, including the United States and Great Britain, the current prevalence of VS is unknown (11). However, in 1994, it was determined that as many as 10,000–25,000 U.S. adults and 4,000–10,000 children were diagnosed as being “awake but not aware” (12, 13). A recent systematic review of prevalence studies on VS revealed a wide variation in outcome of 0.2–6.1 patients per 100,000 members of the general population (14). In a nationwide study in the Netherlands, a prevalence of 0.1–0.2 hospitalized and institutionalized VS/UWS patients per 100,000 members of the general population was indicated (15).

Persistent VS has been defined as the condition remaining 1 month after acute traumatic or non-traumatic brain injury (TBI) and it implies reversibility (12). However, VS which is 3 months after non-TBI or 12 months after TBI to all intents and purposes is regarded as a permanent VS, and it reflects irreversible injury of impaired consciousness. Currently, the official definition of permanent VS is based on the following seven diagnostic criteria: (1) no evidence of awareness of self or environment and an inability to interact with others; (2) no evidence of sustained, reproducible, purposeful, or voluntary behavioral responses to visual, auditory, tactile, or noxious stimuli; (3) no evidence of language comprehension or expression; (4) intermittent wakefulness manifested by the presence of sleep–wake cycles; (5) sufficiently preserved hypothalamic and brain stem autonomic functions to permit survival with medical and nursing care; (6) bowel and bladder incontinence; and (7) variably preserved cranial-nerve reflexes and spinal reflexes (12).

### Development of the VS and Its Diagnosis

**Figure 1:**
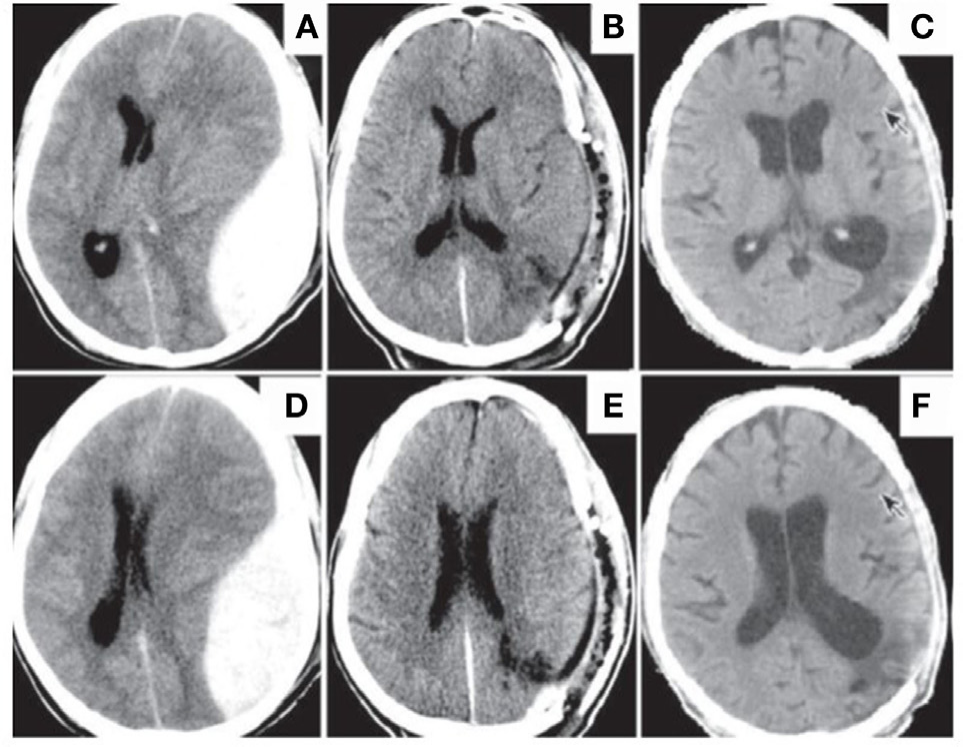
**Traumatic head injury resulting in development of VS with subsequent bilateral cortical atrophy**. Patient was admitted with extradural hematoma **(A,D)**. Evacuation of hematoma relieved brain asymmetry **(B,E)**. However, patient decompensated to a VS with delayed cerebral atrophy observed at 4 months post-op. Note enlarged sulci, particularly in the fronto-temporal lobes **(C,F)**. Reproduced from Louzada et al. (65) with permission.

Typically, VS develops as a consequence of hypoxic-ischemic encephalopathy in which the entire brain is involved, with cardiac/pulmonary arrest, strangulation, head injury, or near drowning being the causative factor (Figure 1). Brain swelling secondary to TBI is also a common cause of this global ischemia. The VS is usually preceded by a coma, with about 1–14% of cases developing clinical indicators of this condition, that include the presence of decorticate posturing, ventilatory dysfunction, and extraneural trauma shortly following the insult (7). Other features suggestive of a vegetative outcome include a poor motor response, advanced age, and an abnormal pupillary reflex. On the other hand, patients in non-traumatic comas display impaired eye opening, altered motor responses, and a loss of the ability to respond adequately to commands after 2 weeks, which together are highly suggestive of VS.

The diagnosis of VS is usually dependent on a number of investigations that include (i) bedside clinical evaluation, (ii) electroencephalographic (EEG) measurements, including event related potentials, a derivate of the EEG, which can be helpful in investigating cognitive processing and possibly conscious awareness in VS patients, and (iii) functional neuroimaging that can include positron emission tomography which provides information on cerebral metabolism and activation of particular brain regions, and functional magnetic resonance imaging, which can also identify brain activity in terms of increased local cerebral blood flow in response to sensory stimulation.

### Cerebral Structures Associated with VS

Results of autopsy studies of patients in VS at the time of death have identified widespread neuronal death throughout the thalamus as the common finding following either anoxia or diffuse axonal injury that produces widespread disruption of white matter connections (16). The severe bilateral thalamic damage after either trauma or anoxia seen in permanent VS is not, however, invariably associated with diffuse neocortical neuronal cell death. Specific thalamic regions exhibit profound neuronal cell death as a consequence of brain trauma injuries, e.g., the central thalamic nuclei that include the intralaminar nuclei and paralaminar nuclei (17). Rostrocaudally, severe disability is reflected in the extent of neuronal cell death, in which severity of dysfunction progresses in the rostrocaudal direction, with the anterior intralaminar and surrounding areas at first exhibiting negative volume change that is linked to moderate disability, while damage to the posterior intralaminar nuclei is associated with increased disability, often seen in both VS and in minimally conscious patients (17). This large-scale neuronal loss in the thalamus is probably a consequence of severe cerebral injury mainly due to diffuse trauma, hypoxia, and other non-specific damage. Direct injury to the central thalamus leads to coma, indicating these cells are an important factor in the regulation of arousal (18). This part of the brain contains circuitry that has a key role to play in the sleep–wake cycle, involving the brain stem associated arousal system (18). It is also innervated by projections from the basal forebrain. Thus, these wiring influences can modulate the level of arousal associated with generalized alertness and variations in cognitive effort, stress, sleep deprivation, and other variables affecting the wakeful state (18, 19). In addition, studies have identified diffuse axonal injury in 70–80% of the studied brains, along with widespread damage to the white matter (20, 21).

### Stem Cell-Mediated Therapeutic Intervention in Patients with VS

#### Endogenous NSCs

Given that patients in VS can spend months, even years in this condition, an important consideration should be whether a stem cell treatment paradigm might be beneficial under these circumstances, taking advantage of the long-term condition of these cases. In recent years, a new approach to the treatment of disease has been developing, one that holds considerable potential for large numbers of individuals in which damage to an organ is presently unrepairable with drugs, surgery, or other forms of therapy. The relatively recent discovery that in selected regions of the brain neurons can be produced during adult life has invigorated both scientists and the public, focusing interest on the possibility of manipulating the development of these endogenous neurogenic populations and utilising them for treating a variety of neurological and neurodegenerative diseases. Studies have demonstrated that neurogenesis occurs throughout life but is mainly limited to the periventricular subventricular zone (SVZ), in which neurons develop that are destined for the olfactory bulb, and the subgranular zone (SGZ) of the hippocampus, in which new neurons are established within the dentate gyrus (22, 23). Neural stem cells (NSCs) are an undifferentiated population of cells residing in the SVZ and SGZ of both the embryonic and adult mammalian brain (24–26). In the adult brain, pathological events, such as TBI (27), stroke (28), seizures (29, 30), and neuroinflammatory states (31), can stimulate neurogenesis, which can have a protective role. Evidence thus far suggests that while these cells show a positive response to the insult, the long-term beneficial effects are limited in nature. On the other hand, the response of these cells can be enhanced via exogenous means and augmentation of endogenous NSCs could therefore be a potential therapy for treating the injured brain. For example, basic fibroblast growth factor or epidermal growth factor can enhance TBI-induced cell proliferation in the hippocampus and the SVZ, and drastically improve cognitive functional recovery (32, 33). In addition, several drugs have shown the potential to enhance neurogenesis and improve cognitive function in experimental TBI. These include statins (34), progesterone (35), erythropoietin (36, 37), and the antidepressant imipramine (38). Such studies lend credence to the notion that increasing the activity of endogenous NSCs may provide an important therapeutic approach for treating patients in VS.

#### Stem Cell Transplantation

Due to the limited repair potential available to the damaged brain, the prospect of neural transplantation represents an important and viable choice for the treatment of injured cerebral tissue (39). In addition to providing a substrate for the regeneration of circuitry lost by the insult, transplanted cells originating from different stem cell sources may also stimulate surviving circuitry to establish new synaptic connections by way of providing trophic support. Potential sources of these cells include adult NSCs, bone marrow cells, umbilical cord cells, and induced pluripotent stem cells (iPSCs).

#### Adult NSCs

In humans, multipotent stem/progenitor cells have been identified and successfully isolated from various regions of adult human brain, including the hippocampus, SVZ, neocortex, and subcortical white matter from neurosurgical resection tissue (40–42). These cells could potentially be used as an autologous cell source for transplantation therapy. Thus far, very few studies have examined the behavior of adult-derived human NSCs in the injured adult CNS. Previous studies have reported that 4% of adult human NSCs survive for 4 months following transplantation in rats with hippocampal damage due to ischemia (43). However, whether these cells become properly integrated with the existing circuitry remains unclear. Another concern as to the potential use of these cells in terms of their translational value relates to the possibility of tumor development, which could have negative ramifications regarding their future use in a clinical setting. On the other hand, use of autologous transplantation as an option, involving the neurosurgical isolation of NSCs from patients with head injury cannot be disregarded and needs to be fully engaged as a viable option. However, whether these cells remain viable for a significant period of time post-surgery while retaining the capacity to improve functional outcome in patients with VS remains an unanswered question that needs to be addressed in the future.

#### Bone Marrow Cells

Mesenchymal bone marrow stromal cells (BMSCs) are a mixture of undifferentiated cells that includes stem and progenitor cells. Their ability for self-renewal and multipotential capability provides for applications in tissue engineering and regenerative medicine. These cells can be readily isolated from bone marrow and grown in culture without ethical and technical concerns. Several studies have reported that transplanted BMSCs accelerate neuroplasticity and facilitate neuronal regeneration and functional recovery (44–47). In addition, they exhibit an ability to enhance the proliferation of endogenous NSCs (48). Such attributes make them an attractive candidate for the treatment of VS, particularly given their low antigenicity due to reduced expression of the major histocompatibility complex (class II) antigens (49). These cells produce high levels of growth factors, cytokines, and extracellular matrix molecules that could have potential neurotrophic or neuroprotective effect in the injured brain. Indeed, studies have demonstrated that the beneficial effects of transplanted BMSCs are attributed to their neurotrophic or neuroprotective effect rather than direct cell replacement (50).

Transplantation of human BMSCs into rats following TBI led to an improvement in sensorimotor and spatial learning along with a reduction in brain injury volume and improved angiogenesis (51, 52). This improved sensorimotor function was evident even when treatment with these cells was delayed by 2 months following brain injury (53), indicative of the long-term viability of these cells and their ability to ameliorate the extent of cerebral damage following administration at later times. This suggests that treatment of cases of VS with these cells has the potential to benefit patients even if treatment is delayed following the insult. In a recent study, 45% of cases of VS treated with autologous BMSCs showed an improvement in consciousness (54). Allogenic transplantation of hematopoietic stem cells (HSCs) from bone marrow has also been promising in the treatment of X-linked adrenoleukodystrophy (ALD), a progressive disease presenting most commonly in males, in which cerebral demyelination due to improper fatty acid metabolism in early childhood can rapidly lead to a VS (55). Autologous treatment of HSCs in combination with gene therapy, involving an introduction of wild-type cDNA of the mutated gene into these cells has provided additional benefits for young children with ALD, in which arrest of cerebral demyelination was achieved (56). Altogether, these findings support the idea that BMSCs provide an important potential for treatment of the injured brain and strongly points to benefits from BMSC treatment, highlighting the notion for future use of these cells in the treatment of VS.

#### Umbilical Cord Blood

**Figure 2:**
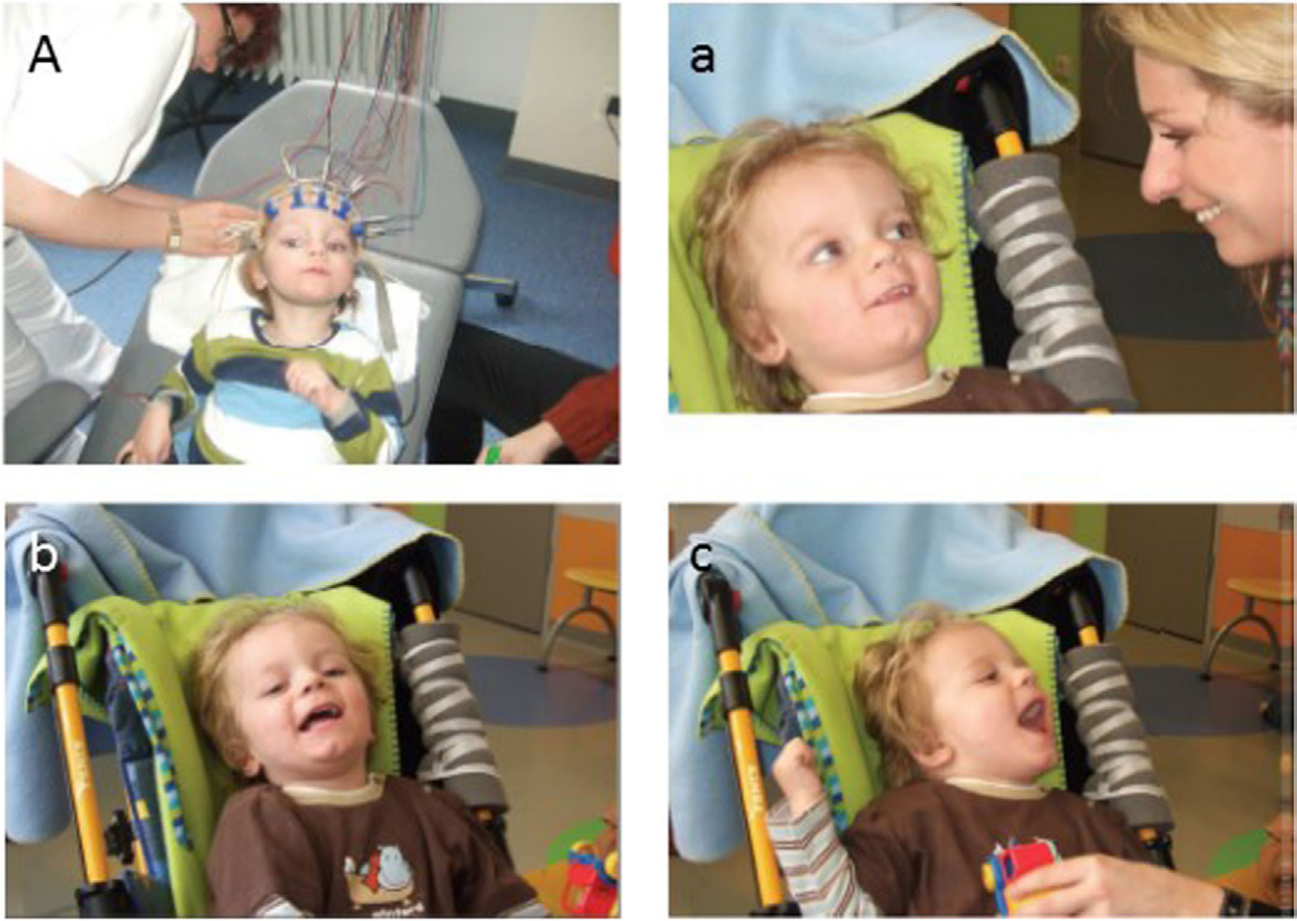
**The patient (L.B.) before and after autologous umbilical cord blood transplantation**. (A) The patient during EEG recording is in a VS 9 weeks after the insult before transplantation of cord blood cells. Note the dilated, unresponsive pupils in spite of bright light from the ceiling. **(a–c)** Two-month follow-up. **(a)** First social smiling of the patient (L.B.) toward his mother and **(b,c)** laughing, when played with, 2 months after transplantation of cord blood cells (i.e., 4 months and 1 week after severe brain damage caused by cardiac arrest). Reproduced from Jensen and Hamelmann (58) with permission.

Several key features make umbilical cord blood an attractive prospect for treatment of VS. Cord blood provides large numbers of HSCs, mesenchymal stem cells, unrestricted somatic stem cells, and embryonic-like stem cells. These cells can be easily harvested at birth without ethical controversy, can be administered with no risk of allograft rejection, and without ethical complications (57). In a landmark study, autologous intravenous cord blood transplantation in a two and a half year old child was shown to considerably improve neurologic function over a period of 40 months following cardiac arrest and development of VS, representing the first reported case of a successful cell therapy treatment of pediatric cerebral palsy (58) (Figure 2). In an earlier report, treatment of a 16-month-old child in VS with cord blood led to lesser though significant clinical improvement over a 6-month period (59). In addition, allogenic cord blood treatment of an 8-year-old boy with ALD prevented neurological deterioration and halted disease progression (60). Notably, one study involving an 8-month-old infant in VS suggests that it may be possible to influence the movement of these cells in the brains of such patients when labeled with paramagnetic iron oxide particles and using an external magnetic field following their transplantation into the ventricular system (61). Such an approach could be useful in helping to direct transplanted stem cells toward the site of a lesion. These findings strengthen the case that it is possible to use stem cells as a viable, major therapeutic strategy for the treatment of patients in VS, one that should be engaged, not ignored.

#### Induced Pluripotent Stem Cells

The development of iPSCs (62), involving somatic cell reprograming, represents an important watershed, providing the potential for novel neural replacement strategies. The main characteristics of iPSCs are unlimited self-renewal and pluripotency (63), defining the ability to differentiate into three germ layers and multilineage cell types. Significantly, a major advantage of the use of patient-specific iPSCs is that it can be safely administered as an autologous treatment without the potential for rejection. Such properties of these reprogramed cells help provide considerable hope that VS and other disease states in which neurodegeneration is a feature might be successfully treated in the future. Indeed, in the 10 years since the seminal work of Takahashi and Yamanaka (62), significant progress has already occurred in deriving iPSCs suitable for clinical use (64).

## Conclusion

For decades, the VS has been considered to represent a severe neurological condition, recovery from which was extremely unlikely, if at all. Patients might remain in this condition for years without much chance for significant recovery. However, recent advances in our understanding of the ability of stem cells to positively influence repair of the damaged brain suggests that the VS may no longer be the largely irreversible maladie of years past. Although in the absence of an established animal model, the question of how stem cell treatment might modify consequences of the VS has remained unaddressed for the most part, a few recent case studies involving the use of these cells have been reported. Their findings suggest that stem cell treatment may offer new hope to effect significant recovery in terms of the level of consciousness, and potentially overall cerebral function. To further our understanding of how this might come about, development of viable animal models of the VS represents an issue that should be pursued in the future, particularly in terms of the potential for stem cell intervention. Currently, no well established *in vivo* models of this condition exist, mainly due to the difficulty of keeping the animal alive for extended periods of time following an insult of this magnitude. A stronger push toward development of such experimental models is likely to pave the way toward identification, and a better understanding, of potential therapeutic interventions that may improve the overall condition of patients in VS over the long term.

## Author Contributions

The author confirms being the sole contributor to this manuscript and approved it for publication.

## Conflict of Interest Statement

The author declares that the research was conducted in the absence of any commercial or financial relationships that could be construed as a potential conflict of interest.
